# 

**Published:** 1851-01

**Authors:** 


### CONTENTS.

### LIBRARY.

A Practical Treatise on Dental Medicine, as connected with the Study of Dental Surgery. By Thos. E. Bond, M. D....... 269

### JOURNAL.

Article I.—Dental Neuralgia. By A. C. Dayton, M. D., Dentist, Vicksburg, Miss........................................ 109

Article II.—Relation between the Dental Organs and the Uterus. By Prof. W. R. Handy, M. D.............................. 127

Article III.—Observations on the Ætiology and Pathology of Odontatrophia. By Chapin A. Harris, M. D.............. 132

Article IV.—Application of Gutta Percha to Dental Purposes,... 145

Article V.—The Percentage System amongst Dentists,.......... 151

Article VI.—Observations on Hare-lip. By Alfred A. Blandy, M. D., Baltimore,.......................................... 155

Article VII.—Successful Operation on, and Treatment of, a Disease of Antrum Highmorianum of the Right Side. By Chas. H. Dubs, Dentist, Natchez,................................ 161

ARTICLE VIII.—Importance of the Preservation of the Teeth. By L. S. Parmly, M. D.................................... 165
ARTICLE IX.—Operations on the Antrum. By R. D. Addington, D. D. S.................................... 169
ARTICLE X.—On the Use of Gutta Percha in Fractures of the Jaw. By H. N. Wadsworth, Georgetown, D. C.......... 171
ARTICLE XI.—Reported Case of Epulis. By R. McLimont, D. D. S. 173

### SELECTED ARTICLES.

ARTICLE XII.—Mechanical Dentistry. By T. L. Buckingham, Dentist, Philadelphia,.................................... 174
ARTICLE XIII—Filling Teeth. By James Taylor, M. D., D. D. S. 182
ARTICLE XIV.—Artificial Teeth on the Atmospheric Principle,... 190
ARTICLE XV.—The Food and the Teeth: Observations on the Inorganic Constituents of the Food of Children, as connected with the Decay of the Teeth, and the Physical Constitution of Women in America. By James Paul, M. D........... 192
ARTICLE XVI.—A Piece of Bone and Teeth, which were dissected from between two Layers of Membrane forming the Walls of an Ovarian Cyst, taken from a Girl after death,............ 218
ARTICLE XVII.—Nasal Polypus,.............................. 221

### EDITORIAL DEPARTMENT.

Progress of Dental Surgery,.................................. 222
Cheap Advertising Dentists—Extraordinary Verdict,............. 224
Another Drill Stock,.......................................... 225
Growth of Hair, Nails, and Secretions of the Body,............. 225
Reinhardt's Adjuster for Fractures of the Lower Jaw,........... 226
To Inventors of Dental Instruments, Chairs, &c................. 226
New Tongue Holder,........................................... 227
Improved Forceps,............................................ 227
Amount of Gold Foil used in the United States for Filling Teeth,.. 228
Odontalgia,.................................................. 228
Omitted Notices,............................................. 228
To Subscribers,.............................................. 228
A Partner Wanted,............................................ 228

